# Supplementary material for: Differential Hsp90-dependent gene expression is strain-specific and common among yeast strains
Source: iScience. 2023 Apr 10;26(5):106635. doi: 10.1016/j.isci.2023.106635 (PMC10149407; doi:10.1016/j.isci.2023.106635)
Supplement: Document S1. Figures S1–S9 [file mmc1.pdf]

iScience, Volume *26*

Supplemental information

Differential Hsp90-dependent gene expression is strain-specific and common among yeast strains

Po-Hsiang Hung, Chia-Wei Liao, Fu-Hsuan Ko, Huai-Kuang Tsai, and Jun-Yi Leu

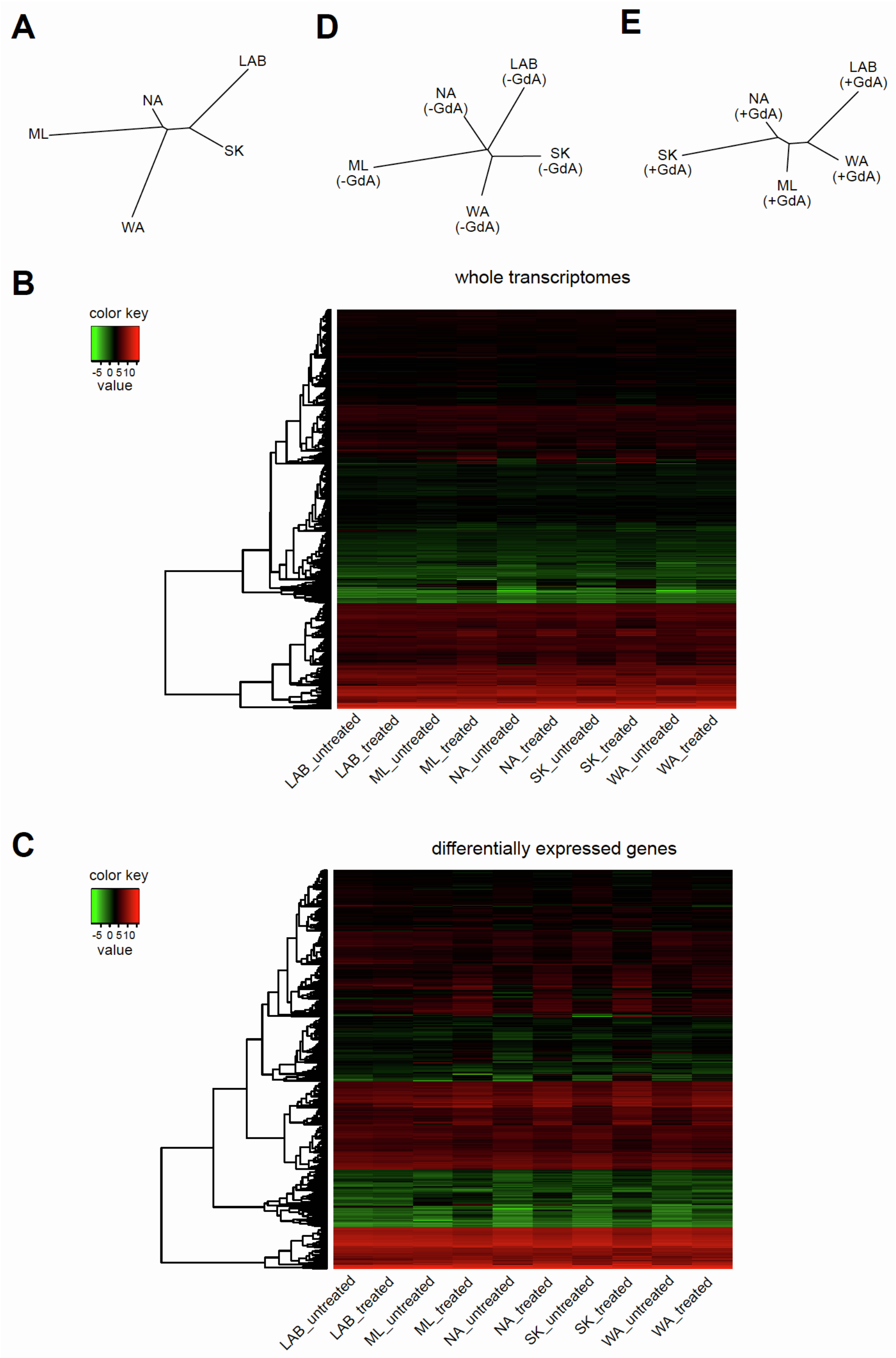

Figure S1. The expression profile distance in the normal condition is similar to the genetic distance among strains, related to Figure 1.

(A) The phylogenetic tree was built based on the variants called from GATK. (B-C) Heatmaps of whole transcriptome profiles (B) and union of differentially expressed genes (C) among 5 strains and 2 conditions (whole transcriptome, n=5,748; differentially expressed genes, n=2,231). The color represents $\log_2$(average TPM+0.001) of each gene in the indicated strain and condition. Genes with 0 TPM were assigned as 0.001 to avoid log transformation errors and they showed as the brightest green color in the heatmaps. Clustering was based on the Euclidean distance of expression profiles. (D) The expression distance among strains is the normal condition. The average TPM value from three biological repeats divided by 1000 was used to calculate the Euclidean distance among strains. (E) The expression distance among strains is the Hsp90-inhibiting condition (50 μM GdA).

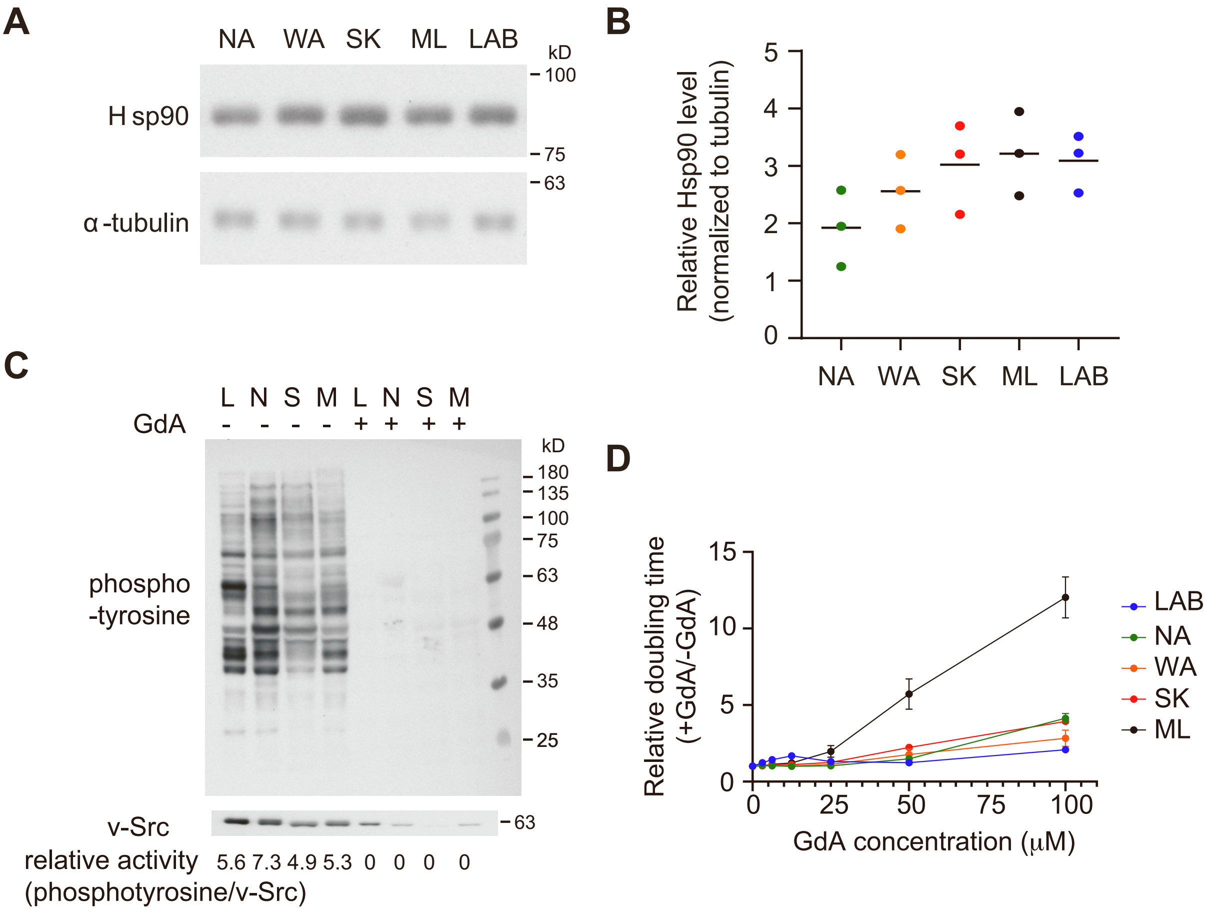

Figure S2. The effects of the GdA treatment on the Hsp90 inhibition and cell growth in different strains, related to Figure 1.

(A) One representing Western blot showing the protein levels of Hsp90 and the internal control, α-tubulin, in different strains. The protein abundance was assessed by Western blotting using antibodies against Hsp90 and tubulin, respectively. (B) Quantification of normalized Hsp90 abundance. The Hsp90 abundance is similar among strains (one-way ANOVA, p=0.1965). (C) The Hsp90 activity is inhibited to a similar level in the tested strains when treated with GdA. To measure the effectiveness of GdA treatment, we performed the v-Src assay to measure the Hsp90 activity [1]. Since the WA strain could not induce the GAL promoter that drives the v-Src expression, only four strains (LAB, NA, SK, and ML) were included in this experiment. Log-phase cells carrying the v-Src construct were grown in the galactose-containing medium for 6 hr with or without 50 μM GdA, and then the v-Src protein abundance and the phosphotyrosine signals were measured (see STAR Methods). The initial expression levels of v-Src varied between strains since individual strains have slight differences in their responses to the galactose induction. Nonetheless, it would not affect our measurement since the tyrosine phosphorylation signals were normalized to the v-Src abundance. After the GdA treatment, the v-Src abundance was drastically reduced and the tyrosine phosphorylation signals were reduced to the background level in all tested strains. (D) The effect of GdA treatments on cell growth. We treated the cells with different concentrations of GdA and measured the doubling time. Only the ML strain exhibited a prolonged doubling time when the GdA concentration is equal to or higher than 50 μM. The relative doubling time (+GdA/–GdA) was plotted.

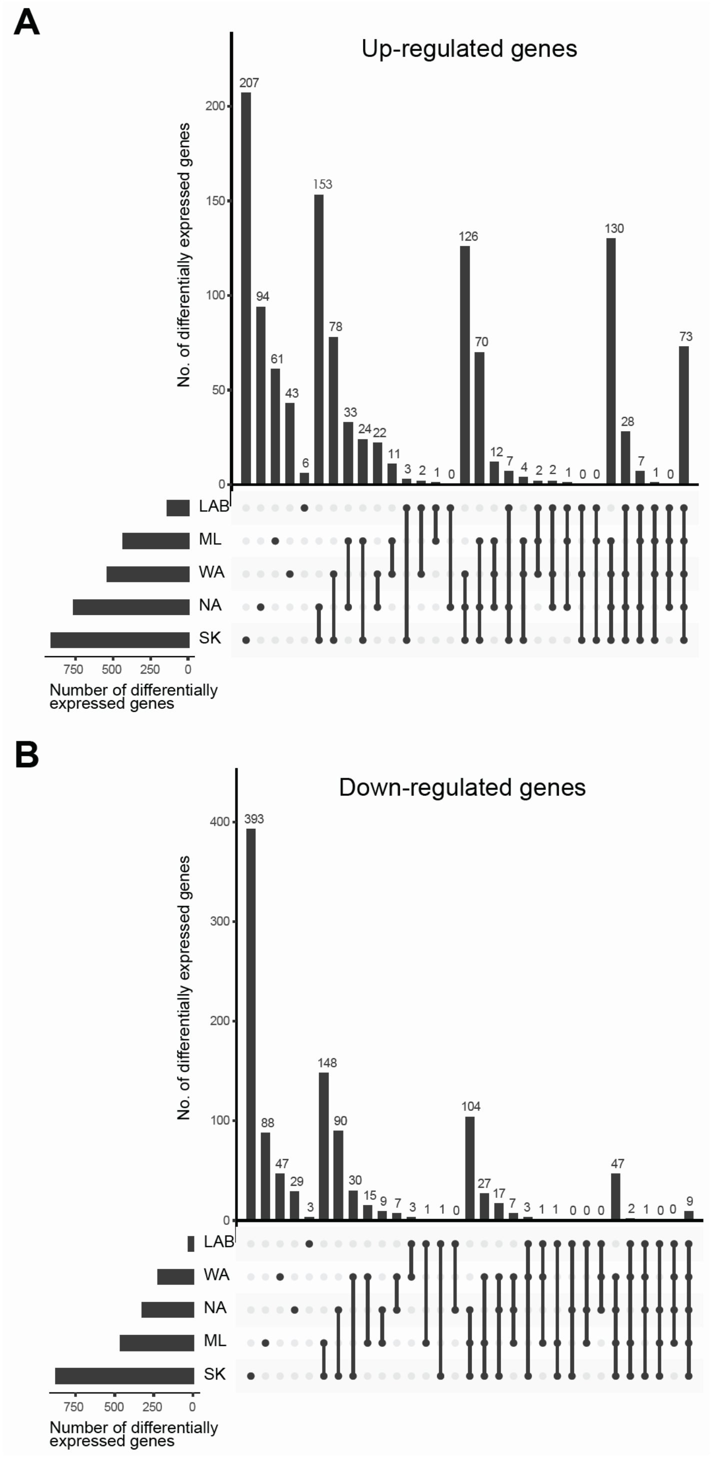

Figure S3. A large proportion of the differentially expressed Hsp90-dependent genes are strain-specific, related to Figure 1.

The transformed Venn diagram was plotted by the R package UpSetR [2]. The total number of differentially expressed genes in each strain is plotted at the bottom-left. The y-axis is the number of differentially expressed genes (+GdA/–GdA) and the x-axis shows the intersection groups linked by the Solid lines. The differentially expressed genes in different intersection groups are mutually exclusive (e.g., if a gene belongs to the LAB-WA-ML-NA-SK group, it will not be counted in the LAB-WA group that only includes the genes shared by LAB and WA but not by others). Up-regulated genes are shown in (A) and down-regulated genes are shown in (B). 239 genes were common up-regulated and 59 genes are commonly down-regulated, i.e., shared by more than three strains.

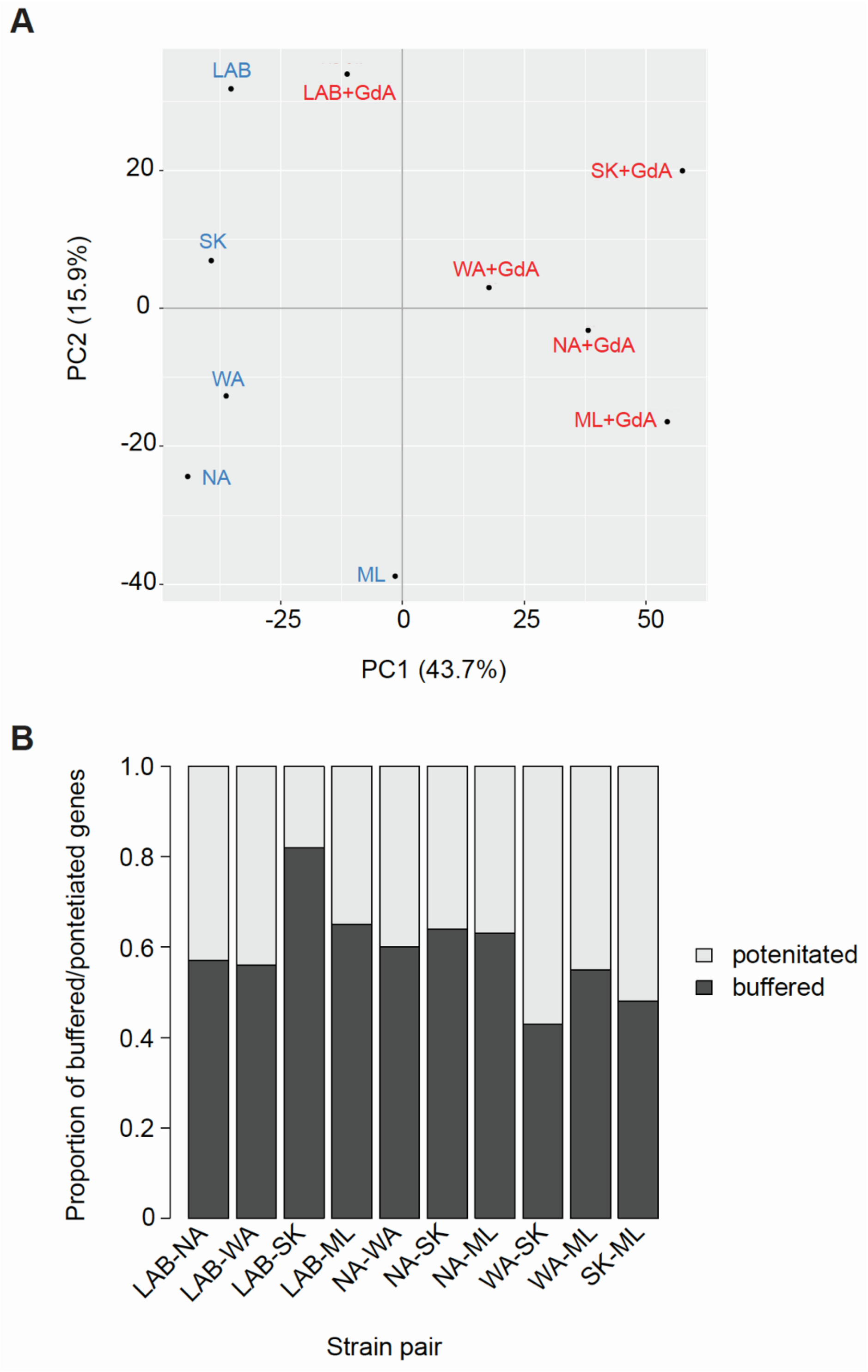

Figure S4. The principal component analysis of expression profiles suggests that the condition-specific effect is the dominant determinant of the observed expression variation, related to Figure 1.
(A) The PC1 explains 43.7% of observed expression variation and it separates most samples by treatment conditions (+GdA and –GdA). The blue color represents the untreated samples (-GdA), while the orange color represents the +GdA condition. (B) Most strain pairs have more buffered genes than potentiated genes. Buffered and potentiated genes were defined as described in Figure 1A and were listed in Table S1H. The proportions of buffered and potentiated genes in each strain pair were plotted.

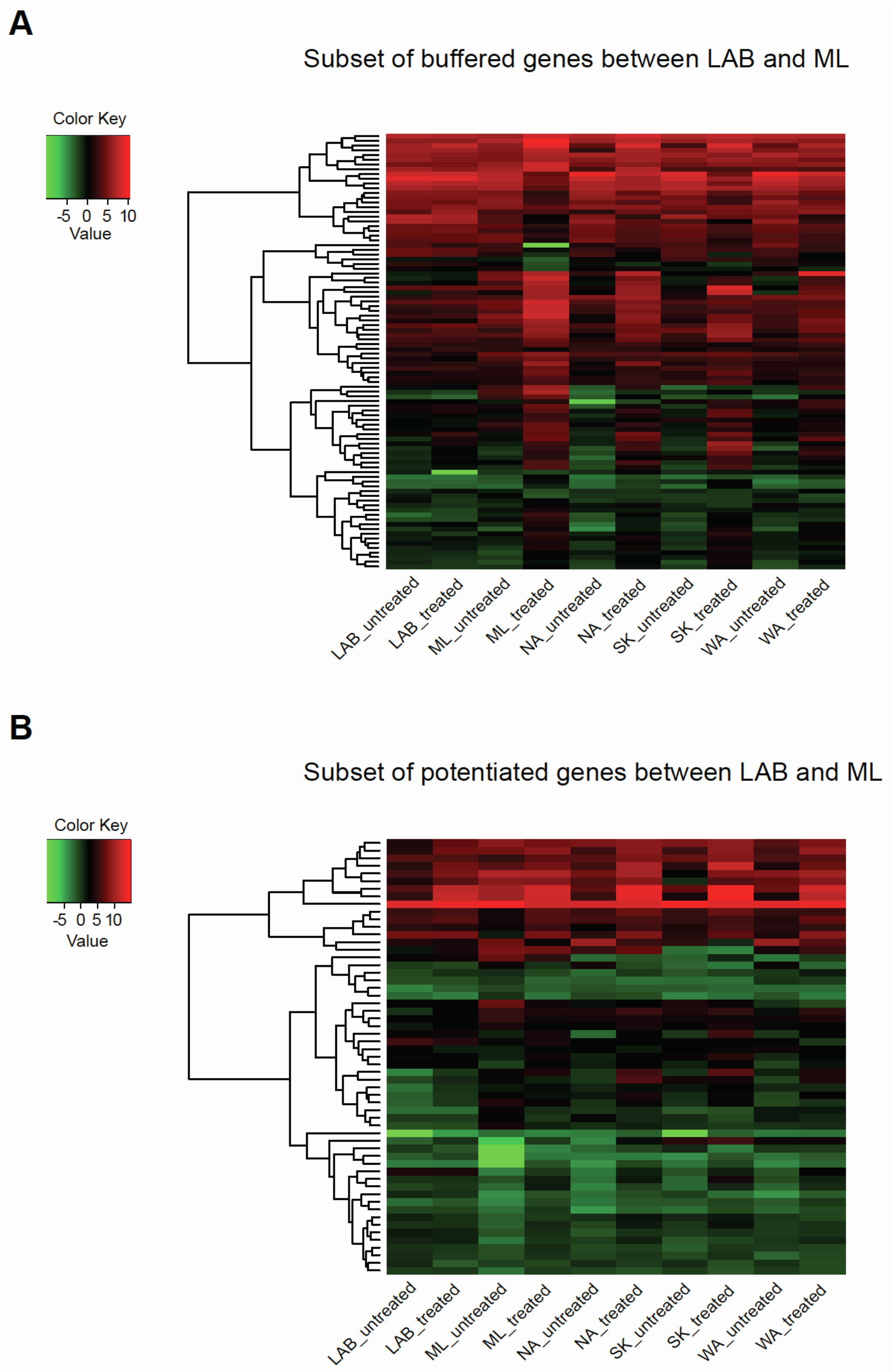

Figure S5. Expression profiles of a subset of buffered and potentiated genes, related to Figure 1.

Heatmaps of buffered (A) and potentiated (B) genes between the LAB and ML strains were plotted based on the $\log_2$(average TPM+0.001) value of each gene in the indicated strain and condition. Clustering was based on the Euclidean distance of expression profiles. The buffered and potentiated genes were defined as explained in Figure 1A.

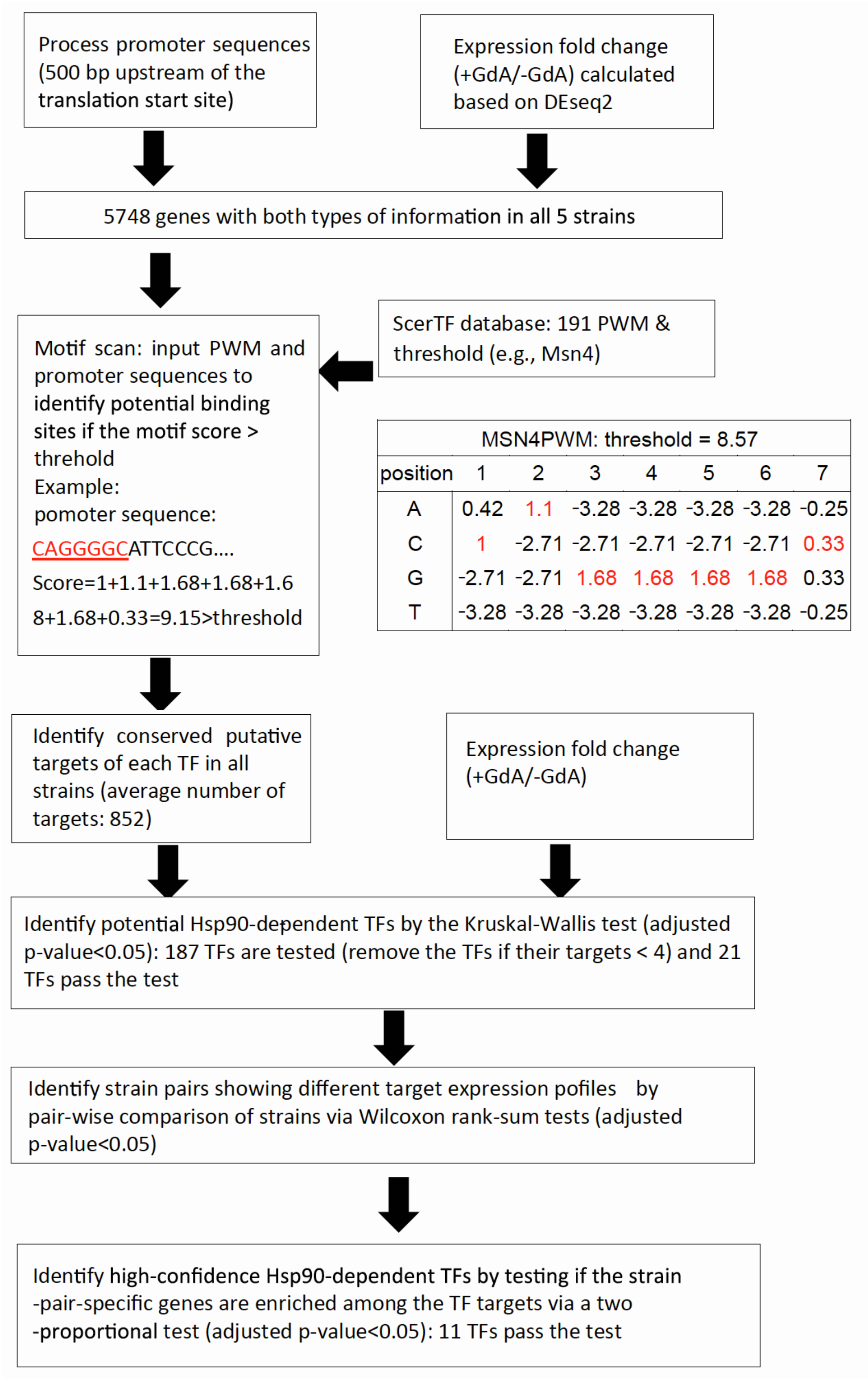

Figure S6. Analysis pipeline for identifying potential Hsp90-dependent strain-specific TFs (related to the method described in sections *Prediction of transcription factor binding sites* and *Identification of candidate TFs*), related to Figure 2.

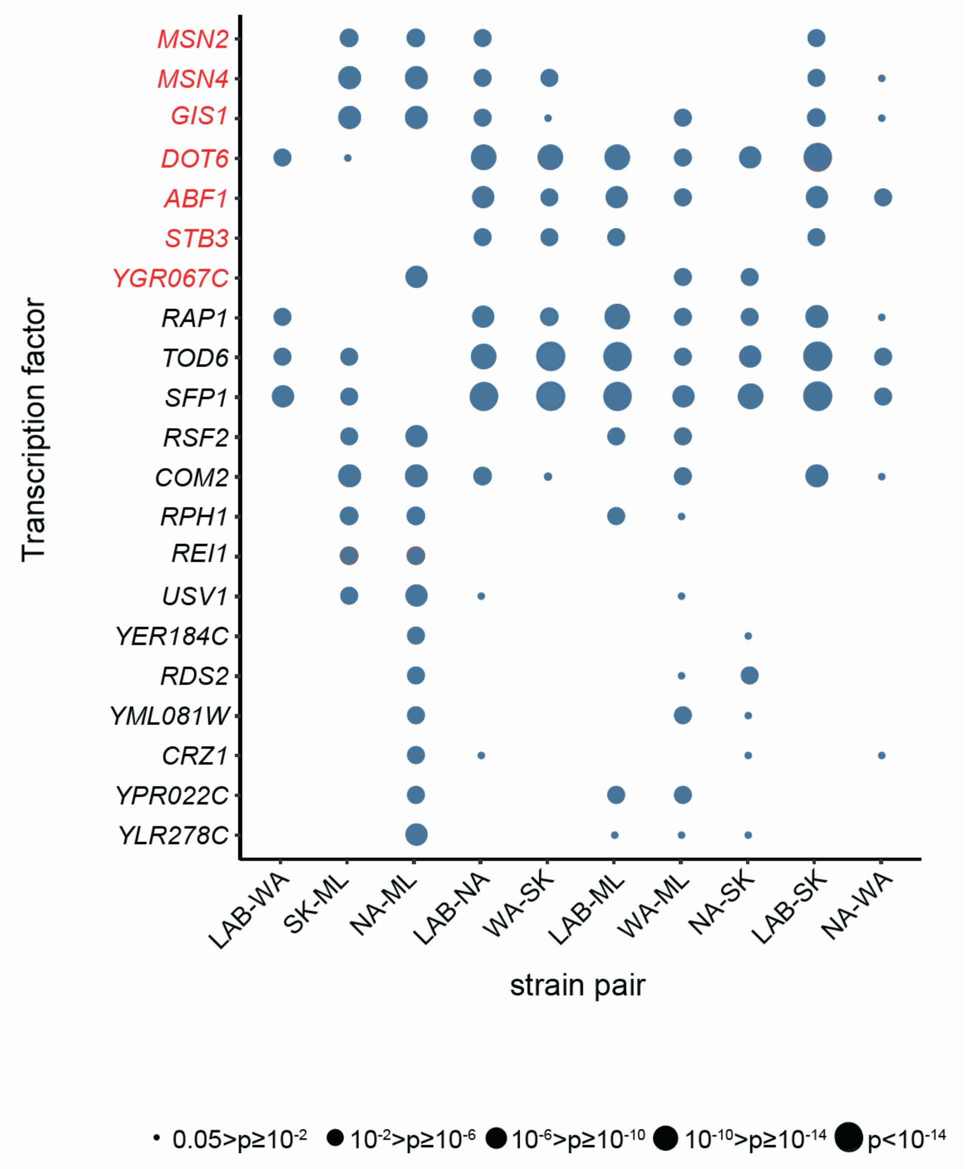

Figure S7. Candidate transcription factors involved in Hsp90-dependent strain-specific regulation, related to Figure 2.

Criteria for selecting candidate TFs were as follows: expression profiles (+GdA/–GdA) of the TF targets should (1) show significant differences among the five strains (Kruskal–Wallis test, adjusted p-value < 0.05), and (2) show significant differences between at least one strain pair (Wilcoxon rank-sum test, adjusted p-value < 0.05). Dot size represents the Bonferroni-adjusted p-value (q-value) from Wilcoxon rank-sum tests. TFs known to physically interact with Hsp90 are labeled in red.

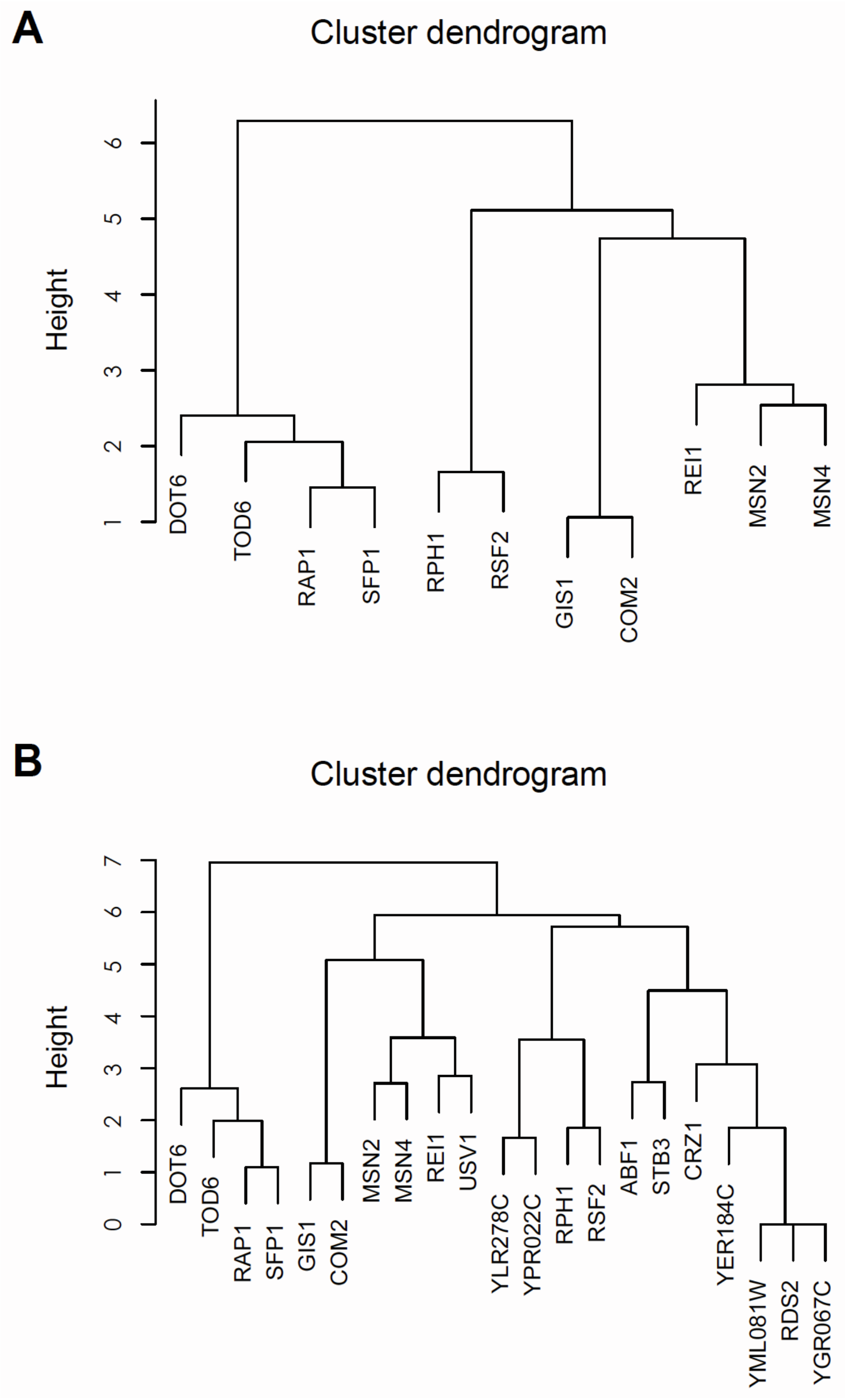

Figure S8. Candidate Hsp90-dependent TFs show different strain-pair-specific response patterns, related to Figure 2.

We clustered the (A) high-confidence Hsp90-dependent TFs and (B) all the candidate Hsp90-dependent TFs based on the Wilcoxon rank-sum test and the enrichment test results (see STAR Methods). The distance matrix is calculated by the Euclidean method and the cluster method is the complete method.

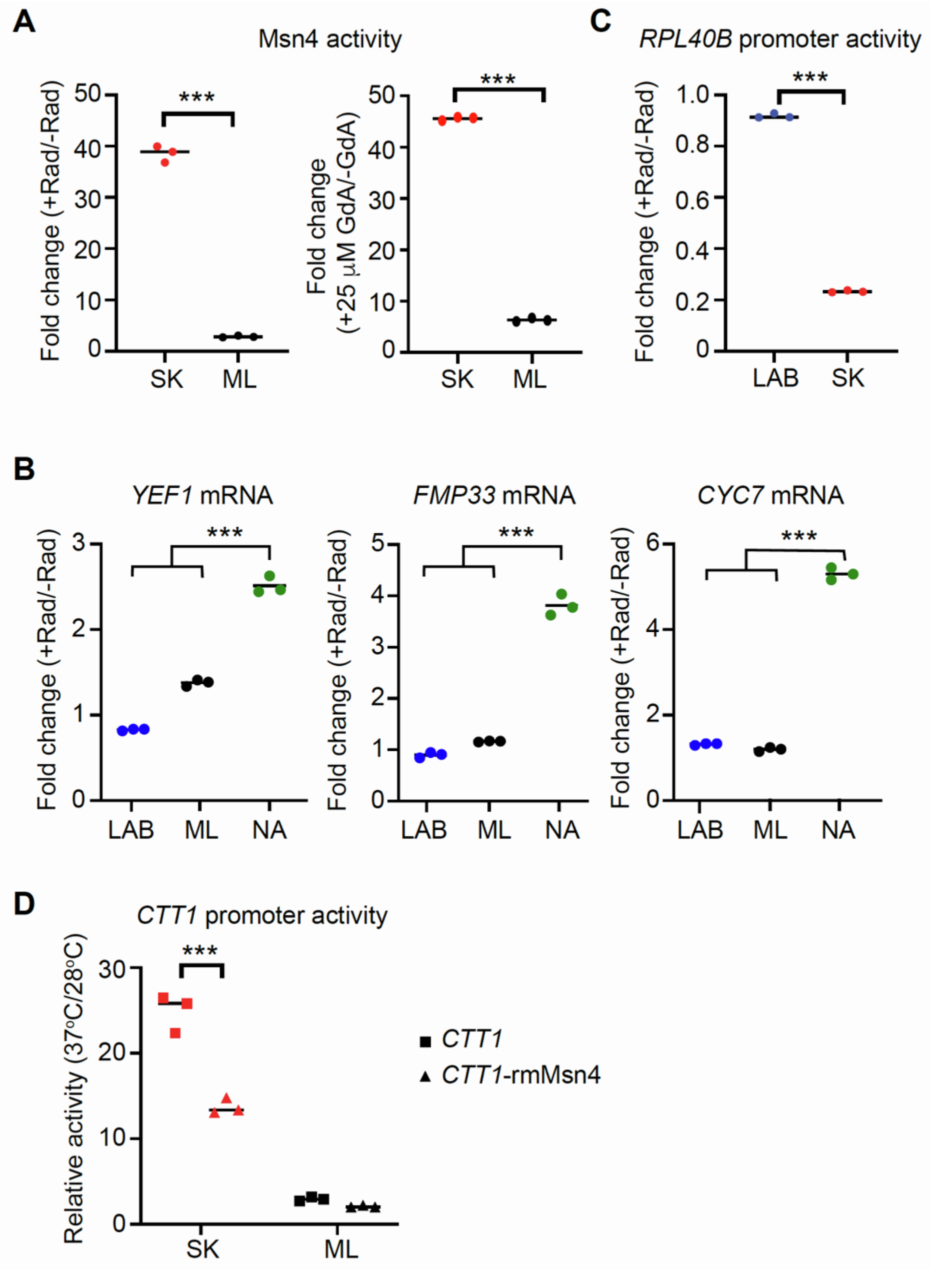

Figure S9. The phenotypes observed in the GdA-treated cells are not GdA-specific and Msn4 has a significant contribution to the SK-specific induction of *CTT1* in the heat stress condition, related to Figures 3, 4 and 6.

(A) The fold changes (25 μM Rad/no Rad and 25 μM GdA/no GdA) of the Msn4 activity are significantly different between SK and ML strains (two-sided unpaired t-test with Welch's correction between strains, $p < 0.001$). Yeast cells were treated with a different Hsp90 inhibitor, radicicol (Rad), or a lower concentration of GdA, and exhibited phenotypes similar to 50 μM GdA-treated cells. We measured the Msn4 activity and *RPL40B* promoter activity in the condition with or without 25 μM radicicol. (B) Gene expression of three Com2 targets, *YEF1*, *FMP33*, and *CYC7*, shows strong NA-specific induction under radicicol treatments (***, adjusted p-value <0.001 based on Tukey's multiple comparisons test). Total RNA was isolated from cells with or without 25 μM radicicol treatments and individual gene expression was measured using q-PCR. (C) The fold changes (25 μM Rad/no Rad) of the *RPL40B* promoter activity are significantly different between SK and LAB strains (two-sided unpaired t-test with Welch's correction between strains, $p < 0.0001$). (D) The induction of *CTT1* was significantly reduced in the SK strain when the Msn4 binding motif was deleted (two-sided unpaired t-test with Welch's correction between strains, $p = 0.0061$). We measured the activities of the full-length and Msn4-motif-deleted (rmMsn4) *CTT1* promoters in both SK and ML strains.

Supplemental references

1. Wayne, N., and Bolon, D.N. (2007). Dimerization of Hsp90 is required for in vivo function: Design and analysis of monomers and dimers. Journal of Biological Chemistry *282*, 35386-35395. 10.1074/jbc.M703844200.
2. Conway, J.R., Lex, A., and Gehlenborg, N. (2017). UpSetR: an R package for the visualization of intersecting sets and their properties. Bioinformatics *33*, 2938-2940. 10.1093/bioinformatics/btx364.
3. Adomas, A.B., Lopez-Giraldez, F., Clark, T.A., Wang, Z., and Townsend, J.P. (2010). Multi-targeted priming for genome-wide gene expression assays. BMC Genomics *11*, 477. 10.1186/1471-2164-11-477.
4. Aghajan, M., Jonai, N., Flick, K., Fu, F., Luo, M., Cai, X., Ouni, I., Pierce, N., Tang, X., Lomenick, B., et al. (2010). Chemical genetics screen for enhancers of rapamycin identifies a specific inhibitor of an SCF family E3 ubiquitin ligase. Nat Biotechnol *28*, 738-742. 10.1038/nbt.1645.
5. Ahmed, K., Carter, D.E., and Lajoie, P. (2019). Hyperactive TORC1 sensitizes yeast cells to endoplasmic reticulum stress by compromising cell wall integrity. FEBS Lett *593*, 1957-1973. 10.1002/1873-3468.13463.
6. Halbeisen, R.E., and Gerber, A.P. (2009). Stress-dependent coordination of transcriptome and translatome in yeast. PLoS Biol *7*, e1000105. 10.1371/journal.pbio.1000105.
